# MicroRNA-1-Mediated Inhibition of Cardiac Fibroblast Proliferation Through Targeting Cyclin D2 and CDK6

**DOI:** 10.3389/fcvm.2019.00065

**Published:** 2019-05-17

**Authors:** Nedyalka Valkov, Michelle E. King, Jacob Moeller, Hong Liu, Xiaofei Li, Peng Zhang

**Affiliations:** ^1^Cardiovascular Research Center, Cardiovascular Institute, Rhode Island Hospital and Alpert Medical School of Brown University, Providence, RI, United States; ^2^Department of Molecular Pharmacology, Physiology and Biotechnology, Brown University, Providence, RI, United States

**Keywords:** cardiac fibroblasts, microRNA-1, cell proliferation, cell cycle regulator, cyclin D2, Cdk6, angiotensin II, TGFβ

## Abstract

MicroRNA-1 (miRNA-1) has been long viewed as a muscle-specific miRNA and plays a critical role in myocardium and cardiomyocytes by controlling myocyte growth and rhythm. We identified that miRNA-1 is expressed in cardiac fibroblasts, which are one of the major non-muscle cell types in myocardium and are responsible for cardiac fibrosis in pathological conditions. In this study, we aimed to investigate the effect and mechanism of action of miRNA-1 on cardiac fibroblast proliferation. Subcutaneous angiotensin II (Ang II) infusion via osmotic minipumps for 4 weeks was used to induce myocardial interstitial fibrosis in male Sprague-Dawley rats. MiRNA-1 expression was significantly down-regulated by 68% in freshly isolated ventricular fibroblasts from Ang II-infused rats than that from control rats. Similar results were obtained in adult rat ventricular fibroblasts that were stimulated in culture by Ang II or TGFβ for 48 h. Functionally, overexpression of miRNA-1 inhibited fibroblast proliferation, whereas knockdown of endogenous miRNA-1 increased fibroblast proliferation. We then identified and validated cyclin D2 and cyclin-dependent kinase 6 (CDK6) as direct targets of miRNA-1 in cardiac fibroblasts using biochemical assays. Moreover, we showed that the inhibitory effects of miRNA-1 on cardiac fibroblast proliferation can be blunted by overexpression of its target, cyclin D2. In conclusion, our findings demonstrate miRNA-1 expression and regulation in adult ventricular fibroblasts, where it acts as a novel negative regulator of adult cardiac fibroblast proliferation that is at least partially mediated by direct targeting of two cell cycle regulators. Our results expand the understanding of the regulatory roles of miRNA-1 in cardiac cells (i.e., from myocytes to a major non-muscle cells in the heart).

## Introduction

Cardiac fibroblasts are one of the most prevalent cell types in the myocardium (1) and play a key role in the maintenance of extracellular matrix in the heart (2). In response to pathological stress or injury, cardiac fibroblasts are activated to proliferate quickly and produce excessive extracellular matrix proteins, which lead to cardiac fibrosis (3). While it has long been viewed as a disease modifier secondary to cardiomyocyte injury, cardiac fibrosis was also suggested to be a primary event in cardiac remodeling (4, 5), for which cardiac fibroblasts increasingly attract attention as direct therapeutic targets (2, 6–8). Thus, it is important to advance our understanding of the regulatory mechanisms that modulate cardiac fibroblast function. Compared to traditional drug targets, microRNAs (miRNAs) offer some novel mechanistic possibilities (9).

MiRNAs are a class of endogenous, evolutionarily conserved, small (~22-nucleotide) non-coding RNAs (10). While in rare cases miRNAs may be able to stimulate mRNA translation (11), they generally negatively regulate gene expression by repressing mRNA translation and/or promoting mRNA degradation by binding to the 3′ untranslated regions (UTRs) of mRNAs (10, 12). MiRNAs are novel regulators in essentially all biological processes and are involved in many diseases (13). In the heart, miRNAs are recognized as important regulators in both cardiac development and disease. Dysregulation of miRNAs has been associated with many cardiac diseases, bringing new insights into the cellular and molecular processes that drive cardiac pathology (14). To date, most studies have focused on miRNAs in cardiac myocytes and their critical role in regulating myocyte hypertrophy, contractility, and arrhythmogenesis [reviewed in (15–17)]. In contrast, a few miRNAs have been studied in cardiac fibroblasts and accumulated evidence suggest that they play an important role in regulating cardiac fibroblast signaling and function [reviewed in (18)]. For example, miRNA-21 was shown to regulate fibroblast survival by mediating MAPK signaling via sprouty homolog 1 (5); miRNA-29 targets extracellular matrix proteins including collagen (19); miRNA-30 targets connective tissue growth factor (20).

MiRNA-1 is evolutionarily conserved across species and is encoded by distinct genes on chromosomes 20 and 18 in the human genome and chromosomes 18 in the rat genome. In the heart, miRNA-1 has been extensively studied in cardiac myocytes and was reported to play a critical role in controlling myocyte growth, rhythm, and cell fate (21). Although it is highly expressed in cardiac and skeletal muscle cells, miRNA-1 has also been found to be expressed and play a role in other cell types and organs. For example, miRNA-1 has been reported in human retinal microvascular endothelial cells and human umbilical vein endothelial cells, where it is down-regulated by high glucose exposure and targets endothelin-1 (22). MiRNA-1 is also recognized as a tumor suppressive miRNA (e.g., in hepatoma and prostate cancer), and its level is further reduced in distant metastasis (23, 24). These findings from non-muscle cells indicate that miRNA-1 is more widely expressed than originally believed (21), although expression levels vary.

Using adult rat ventricular fibroblasts, we found that miRNA-1 is also expressed in cardiac fibroblasts. Giving the critical importance of miRNA-1 in cardiomyocytes and myocardium, investigation of the regulatory effects of miRNA-1 in cardiac fibroblasts became necessary. In this study, we tested the hypothesis that miRNA-1 has a regulatory role in cardiac fibroblast signaling and function with a focus on the regulation of cardiac fibroblast proliferation. Our findings demonstrate miRNA-1 expression changes in adult ventricular fibroblasts and show that miRNA-1 acts as a novel negative regulator of adult cardiac fibroblast proliferation that is at least partially mediated by direct targeting of two cell cycle regulators.

## Materials and Methods

This study was carried out in accordance with the recommendations of the Guiding Principles in the Care and Use of Vertebrate Animals in Research and Training. The protocol involving animals was approved by the Institutional Animal Care and Use Committee of Rhode Island Hospital. All efforts were made to minimize suffering.

### Chronic Ang II Infusion Model

Male Sprague-Dawley rats (5–6 weeks old) were anesthetized with isoflurane mixed with oxygen. Osmotic minipumps (Alzet, Cupertino, CA; models 2002) were used for delivery of Ang II (555 ng·kg^−1^·min^−1^) or 0.9% saline after subcutaneous implantation. The minipumps were replaced after 2 weeks for a total of 4 weeks Ang II infusion. After surgery, the animals received regular chow with 0.4% KCl in drinking water to compensate for loss of potassium as a result of an aldosterone-induced increase in the Na^+^/K^+^ ATPase expression. At the end of the experiments, hearts were removed for subsequent histology analysis and myocyte/fibroblast isolations.

### Echocardiographic Measurements

Transthoracic echocardiography was performed using a Vevo2100 (Fujifilm VisualSonics, Toronto, Canada) echocardiography system with a MS250 probe. Animals were lightly anesthetized using 3% isoflurane for induction and 1.5–2% isoflurane via nose cone for maintenance. Body temperature was kept at 37°C using a heating lamp. Electrocardiogram, respiration, and rectal temperature were continuously monitored with an integrated physiology platform (Fujifilm VisualSonics). 2D images in the left ventricle (LV) parasternal long axis views and M-mode images in the short-axis views at the mid-papillary level were obtained to assess LV dimensions and wall thickness. LV inflow velocities (E and A wave velocities) were interrogated by conventional pulse-wave Doppler from the apical four chamber view with the sample volume placed at the tip of the mitral valve leaflets. The mitral annulus longitudinal velocities (E′ and A′) were determined by pulse-wave tissue Doppler from the apical four chamber view with the sample volume placed at the septal side of the mitral annulus. Measurements were averaged from three consecutive beats during expiration.

### Ventricular Fibrosis Assessments

Heart tissues were formalin fixed and paraffin-embedded. Fibrosis was assessed on ventricular cross sections that were deparaffinized, rehydrated and stained with Picrosirius Red (25). Entire cross-sections were scanned using an Aperio Scanscope® CS System (Leica Biosystems, Buffalo Grove, IL) and analyzed using ImageJ software. Briefly, bright field images were first converted to gray scale using a RGB stack command to split the image into red, green and blue channels. The green channel, which has the best separation, was then used to isolate and measure red-stained collagen by applying a color thresholding algorithm. This segmented and quantified the area of fibrosis within an image. Thresholding of the red channel was then applied to determine total tissue area without empty spaces. After obtaining the individual components and correcting the images for the areas to be excluded, the algorithm measured the areas of all components in pixels (25). The percentage of fibrosis (Sirius Red positive area/total tissue area × 100) was then calculated.

### Isolation and Culture of Adult Rat Ventricular Fibroblasts and Myocytes

Adult male rat ventricular cells were isolated as previously described (26). In brief, hearts were quickly excised from male Sprague-Dawley rats (5–7 weeks-old) under Ketamine/Dexmedetomidine anesthesia, retrogradely perfused for 2 min with Krebs-Henseleit bicarbonate (KHB) buffer containing 118 mM NaCl, 4.7 mM KCl, 1.2 mM MgSO_4_, 1.2 mM KH_2_PO_4_, 25 mM NaHCO_3_, 11 mM glucose, and 8.4 mM HEPES at 37°C, and then switched to enzyme *buffer 1* [KHB buffer containing 0.3 mg/ml collagenase II (Worthington, Lakewood, NJ), 0.3 mg/ml hyaluronidase (Sigma, St. Louis, MO), and 50 μM CaCl_2_]. After 20 min perfusion, the ventricular tissue was cut into small pieces and further digested at 37°C for 18 min in enzyme *buffer 1* supplemented with increased CaCl_2_ (500 μM), trypsin IX (0.6 mg/ml; Sigma), and deoxyribonuclease (0.6 mg/ml; Sigma). The cell suspension was then filtered (200 μm) into 10 ml of DMEM/F12 (Life Technologies, Carlsbad, CA) with 10% fetal bovine serum (FBS), 100 U/ml penicillin, and 100 mg/ml streptomycin (complete medium) and centrifuged for 2 min at 20 *g*. The pelleted myocytes were resuspended in washing medium and the rod-shaped myocytes were concentrated by going through 6.5% bovine serum albumin (BSA) solution, followed by plating into laminin-coated 6-well dishes in Medium 199 supplemented with 2 mg/ml BSA, 2 mM L-carnitine, 5 mM creatine, 5 mM taurine, 0.1 μM insulin [ACCTI “defined culture medium” (27)]. The supernatant that was removed from the pelleted myocytes was centrifuged again for 5 min at 800 *g*. The resulting fibroblast pellet was resuspended in complete medium and pre-plated for 2 h, after which the medium was changed to remove unattached or loosely attached cells. The high purity of our fibroblast preparations was previously characterized (26): the absence of adult cardiomyocytes that can be easily identified by their rod shape and the absence of endothelial cells and vascular smooth muscle cells by negative in staining for their respective markers, von Willebrand factor and smooth muscle myosin. All fibroblast experiments were performed on Passage 1 (P1) cells from normal rats, with exception that freshly isolated fibroblasts from Ang II infused rats and control rats were used to determine the miRNA-1 expression upon fibroblast activation *in vivo*. DMEM/F12 supplemented with 10 μg/ml insulin, 5.5 μg/ml transferrin, and 5 ng/ml sodium selenite (ITS, Sigma) was used as serum-free medium where indicated.

### Analysis of miRNA-1 Expression

Total RNA was isolated using mirVana miRNA isolation kit or Trizol (Life Technologies). The expression levels of mature miRNA-1 were measured using TaqMan MiRNA-1 Assays according to the manufacturer's instructions (Life Technologies). After reverse transcription with TaqMan MiRNA Reverse Transcription Kit (Life Technologies), cDNA samples were amplified using pre-designed FAM-labeled TaqMan miRNA-1 probes together with the TaqMan Universal PCR Master Mix (Life Technologies). PCR cycling was performed using ABI 7900HT at 50°C for 2 min and 95°C for 10 s, followed by 95°C for 15 s and 60°C for 1 min for a total of 40 cycles. Each sample was assayed in duplicate in two independent PCR reactions and normalized to U6 expression. Samples without template during PCR served as negative controls. Assay results were analyzed using sequence detecting system software (SDS version 2.3; Life Technologies).

### Real-Time PCR for mRNA Expression

Reverse-transcribed (TaqMan Reverse Transcription reagents, Life Technologies) RNA samples from fibroblasts were subjected to real-time PCR using FAM-labeled TaqMan probes for atrial natriuretic factor (ANF), cyclin D2, CDK6, and 18S and universal PCR master mix according to the manufacturer's instructions (Life Technologies). Each sample was assayed in duplicate in two independent PCR reactions and normalized to 18S expression. Samples without template during PCR served as negative controls. PCR cycling was performed at 95°C for 10 min, followed by 95°C for 15 s and 60°C for 1 min for a total of 40 cycles using ABI 7900HT. Assay results were analyzed using sequence detecting system software (SDS version 2.3; Life Technologies).

### Overexpression of miRNA-1 or Cyclin D2 by Adenoviral Gene Transfer

Adenovirus encoding the stem-loop sequence of miRNA-1-2 was generated previously (28). Adenovirus encoding HA-tagged cyclin D2 was a gift from Dr. Jun Sadoshima (Rutgers New Jersey Medical School). Empty adenovirus (Ad-Ctr) served as controls. Fibroblasts were cultured in complete medium and infected the next day. To ensure comparable multiplicity of infection (MOI) between experiments, a representative well from each experiment was trypsinized, followed by cell counting. Appropriate amounts of adenovirus in DMEM/F12 were added to each well immediately after medium aspiration.

### MiRNA-1 Knockdown by Antagomir Transfection

Chemically modified, sequence-specific miRNA-1 antagomir (Life Technologies) was used to knockdown endogenous miRNA-1. A negative control miRNA antagomir with no homology to any known mammalian gene served as control (Life Technologies). Fibroblasts were cultured in complete medium and subjected to antagomir transfection the next day using siPORT NeoFX transfection agent (Life Technologies) diluted in OPTI-MEM (Life Technologies). After 10 min of incubation at RT, 75 nM antagomirs were added and incubated for another 10 min. The transfection complexes were then dispensed onto the cells right after medium change with antibiotic free DMEM/F12 containing 2% FBS.

### MTS Cell Proliferation Assays

Fibroblast proliferation was assessed using CellTiter 96^®^ AQ_ueous_ One Solution Cell Proliferation Assay kit (Promega, Madison, WI). After miRNA-1 manipulation, fibroblasts were plated into 96-well plate at a density of 20,000 cells/well. After incubation in serum-free medium for 24 h, cells were treated with TGFβ1 (10 ng/ml) for 48 h, followed by adding 20 μl/well of CellTiter 96^®^ AQ_ueous_ One Solution Reagent into each well. The plates were incubated for up to 4 h at 37°C. Absorbance at 490 nm was measured using a plate reader.

### Western Blot Analysis

Fibroblasts were lysed for 30 min at 4°C in 1x lysis buffer (Cell Signaling Technology, Danvers, MA) containing a protease inhibitor cocktail (Roche, Indianapolis, IN) or a protease/phosphatase inhibitor cocktail (Cell Signaling Technology). Equal amounts of protein per lane were separated on 10% SDS-polyacrylamide (Tris/glycine) gels and transferred to nitrocellulose membranes. After transfer, the membranes were stained with Ponceau S, blocked in phosphate-buffered saline (PBS) containing 5% non-fat dry milk or tris-buffered saline (TBS) containing 5% BSA, and probed with antibodies against cyclin D2 (Rabbit monoclonal antibody, Cat#3741S, Cell Signaling Technology, RRID:AB_2070685, Dilution 1:1,000), cyclin dependent kinase 6 (CDK6, Rabbit polyclonal antibody, Cat#sc-177, Santa Cruz Biotechnology, Santa Cruz, CA, RRID:AB_631225, Dilution 1:1,000), CDK4 (Rabbit polyclonal antibody, Cat#sc-260, Santa Cruz Biotechnology; RRID:AB_631219, Dilution 1:1,000), and phospho-retinoblastoma protein (pRb^ser780^, Rabbit polyclonal antibody, Cat#9307, RRID:AB_330015 and pRB^ser795^, Rabbit polyclonal antibody, Cat#9301, RRID:AB_10830074, Cell Signaling Technology; Dilution 1:1,000, respectively), Proliferating Cell Nuclear Antigen (PCNA, Mouse monoclonal antibody, Cat#sc-25280, Santa Cruz Biotechnology, RRID:AB_628109, Dilution 1:1,000), GAPDH (Rabbit monoclonal antibody, Cat#2118, Cell Signaling Technology, RRID:AB_561053, Dilution 1:1,000). After washing, the membranes were incubated with appropriate peroxidase-coupled secondary antibodies. Proteins of interest were visualized by chemiluminescence (Thermo Scientific, Rockford, IL). Quantitative densitometry was performed using ImageJ software.

### 3′-UTR Luciferase Reporter Assays

pmirGLO Dual-Luciferase miRNA Target Expression Vector (Promega) was used according to the manufacturer's instructions. Oligonucleotide pairs that contain the predicted binding site of miRNA-1 on the 3′-UTR of the putative target (wild type), a mismatched seed sequence (mutation), and a deletion of the seed sequence (deletion) were synthesized (Integrated DNA Technologies, Coralville, IA) and ligated between the *PmeI* and *XbaI* restriction sites of the pmirGLO vector, respectively. The sequences of oligonucleotide pairs are described in Table 1. The authenticity and orientation of the inserts were confirmed by DNA sequencing. Fibroblasts were cultured in complete medium and infected with Ad-miRNA-1 or Ad-Ctr. The next day, fibroblasts were transfected with the plasmid constructs (0.5 μg) that contain wild type, mutation, or deletion of the 3′-UTR binding site of the putative target using Lipofectamine 2000 (Life Technologies) in 12-well plates as per manufacturer's instructions. After 48–72 h, cells were harvested and luciferase activities were measured using the Dual-Luciferase Reporter assay system (Promega) and a DLR-ready luminometer. Normalized firefly luciferase activity (firefly luciferase activity/Renilla luciferase activity) from each construct was compared.

**Table 1:** Sequences of oligonucleotide pairs containing the predicted miRNA-1 binding sites of the putative targets for 3′-UTR luciferase reporter assays.

**Gene name**	**Sequence of oligonucleotide pairs (5**^****′****^**-3**^****′****^**)**		
Cyclin D2	WT	Sense	*AAAC*CCACAGTTTAATGTTGTTATAAAC**CATTCCA**CTAGAAAAGC*T*
		Antisense	*CTAGA*GCTTTTCTAG**TGGAATG**GTTTATAACAACATTAAACTGTGG*GTTT*
	MUT	Sense	*AAAC*CCACAGTTTAATGTTGTTATAAAC***ATCGATT***CTAGAAAAGC*T*
		Antisense	*CTAGA*GCTTTTCTAG***AATCGAT***GTTTATAACAACATTAAACTGTGG*GTTT*
	DEL	Sense	*AAAC*CCACAGTTTAATGTTGTTATAAACCTAGAAAAGC*T*
		Antisense	*CTAGA*GCTTTTCTAGGTTTATAACAACATTAAACTGTGG*GTTT*
CDK6	WT	Sense	*AAAC*GTCCCAGGTTAAAAAACAGACAA**ACATTCCA**GGCTGCTG*T*
		Antisense	*CTAGA*CAGCAGCC**TGGAATGT**TTGTCTGTTTTTTAACCTGGGAC*GTTT*
	MUT	Sense	*AAAC*GTCCCAGGTTAAAAAACAGACAA***AATCGATT***GGCTGCTG*T*
		Antisense	*CTAGA*CAGCAGCC***AATCGATT***TTGTCTGTTTTTTAACCTGGGAC*GTTT*
	DEL	Sense	*AAAC*GTCCCAGGTTAAAAAACAGACAAAGGCTGCTG*T*
		Antisense	*CTAGA*CAGCAGCCTTTGTCTGTTTTTTAACCTGGGAC*GTTT*

*Nucleotides predicted to be the corresponding seed sequences of miRNA-1 are shown in bold and underlined. Mismatched nucleotides in mutation version are in bold italics. Overhangs of restriction enzyme sites are in italics. WT, wild type; MUT, mutation; DEL, deletion*.

### Statistical Analysis

The statistical analysis was performed using the GraphPad Prism Software (San Diego, California). Data are expressed as means ± SEM for *n* determinations. Statistical differences were assessed using unpaired two tailed Student's *t*-test or two-way ANOVA for comparison of individual means, followed by Bonferroni *post-test*. A *P-*value < 0.05 was considered statistically significant.

## Results

### MiRNA-1 Is Expressed in Adult Ventricular Fibroblasts and Down-Regulated Upon Fibroblast Activation *in vivo* and *in vitro*

**Figure 1:**
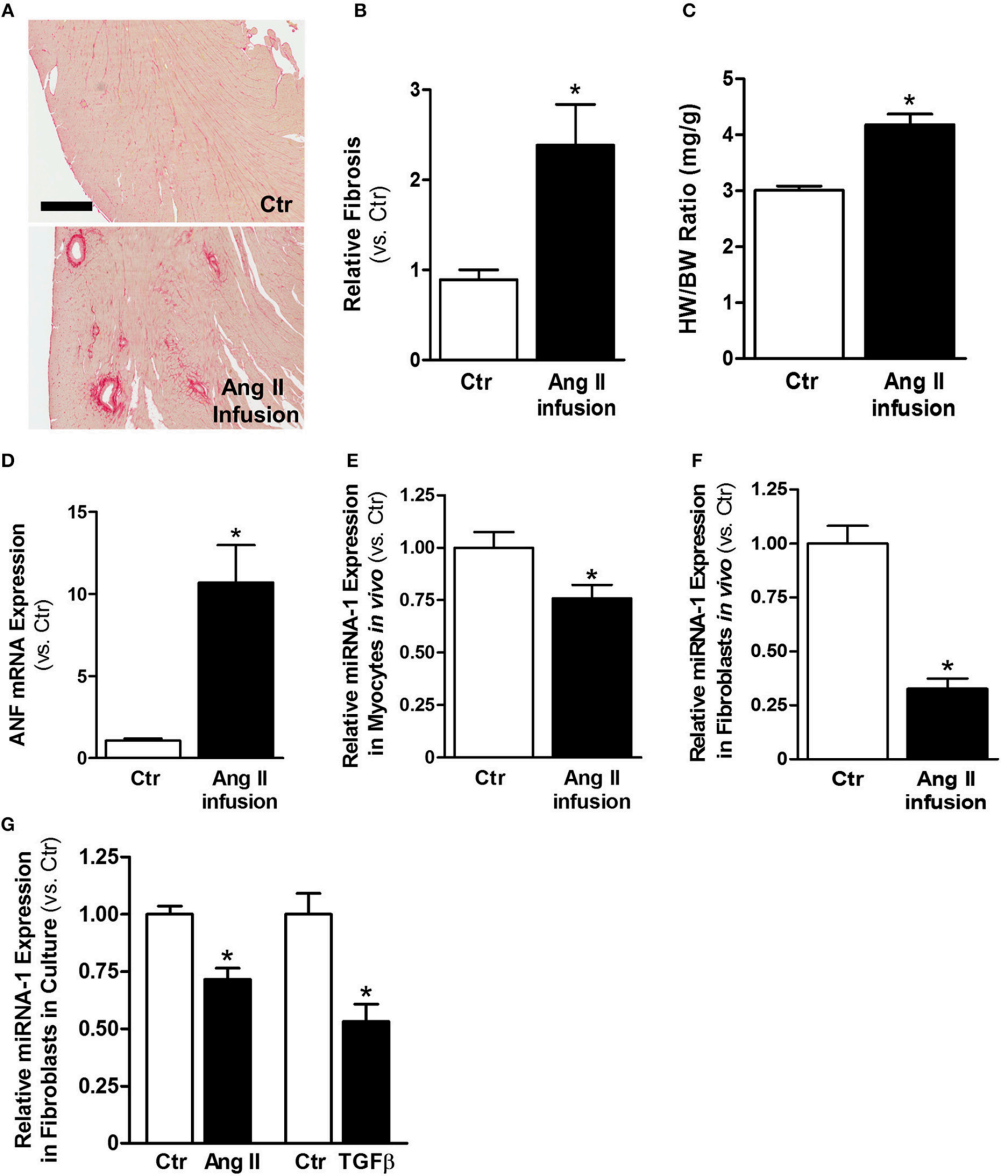
MiRNA-1 is down-regulated upon cardiac fibroblast activation *in vivo* from subcutaneous Ang II-infused hearts and *in vitro* upon Ang II or TGFβ stimulation. **(A)** Representative images of Picrosirius Red staining from free wall (cross sections) of left ventricles of control (saline) and Ang II-infused hearts. Scale bar: 500 μm. **(B)** Quantitation of Picrosirius Red staining in entire ventricular cross-section area. *n* = 5 rats each. Data are expressed as relative changes to Ctr. ^*^P < 0.05 vs. Ctr. **(C)** Heart weight (HW) to body weight (BW) ratios. *n* = 5–6 rats each. ^*^ *P* < 0.05 vs. Ctr. **(D)** Real-time PCR analysis of ANF mRNA expression in freshly isolated cardiac myocytes from rats subjected to subcutaneous saline (Ctr) or Ang II infusion. Data are normalized to 18S and expressed relative to controls. *n* = 8–9 rats each, ^*^P < 0.05 vs. Ctr. **(E,F)** Real-time PCR analysis of miRNA-1 expression in freshly isolated cardiac myocytes and cardiac fibroblasts from rats subjected to subcutaneous saline (Ctr) or Ang II infusion for 4 weeks. Data are normalized to U6 and expressed relative to controls. *n* = 7 rats each. ^*^ *P* < 0.05 vs. Ctr. **(G)** Real-time PCR analysis of miRNA-1 expression in adult rat ventricular fibroblasts (P1) that were treated with Ang II (1 μM) or TGFβ1 (10 ng/ml) for 48 h. *n* = 5–6 each. Data are normalized to U6 and expressed relative to respective controls. ^*^P < 0.05 vs. respective Ctr.

Ang II is a well-known profibrotic factor in many mammalian species, including humans (29), and acts via its downstream factor TGFβ (30). Subcutaneous Ang II infusion via osmotic minipumps was used to induce myocardial interstitial and perivascular fibrosis, in which activated cardiac fibroblasts play a key role. As shown in Figures 1A,B, Ang II infusion for 4 weeks induced interstitial and perivascular fibrosis in myocardium. Cardiac hypertrophy was also developed in Ang II-infused hearts compared to control hearts, as indicated by increases in heart weight to body weight ratios (Figure 1C) and ANF mRNA expression in myocytes (Figure 1D). We also performed transthoracic echocardiography to assess left ventricle (LV) morphology and function. Compared to controls, subcutaneous Ang II infusion for 4 weeks (Table 2) increased LV wall thickness and computed LV mass, and reduced LV end systolic and diastolic volumes, which also indicate development of LV hypertrophy. The increased E/E′ suggests potential LV diastolic dysfunction, while LV systolic function seems still normal. Using this model, we first examined the expression of miRNA-1. As expected, miRNA-1 was significantly down-regulated by 25% in hypertrophied myocytes freshly isolated from Ang II infused hearts than control hearts (Figure 1E). Importantly, we found that miRNA-1 expression was significantly down-regulated by 68% in freshly isolated ventricular fibroblasts that were activated by Ang II infusion *in vivo* for 4 weeks (Figure 1F).

**Table 2:** Echocardiographic measurements from rats subjected to subcutaneous saline (Control) or Ang II infusion for 4 weeks.

**Echo parameters**	**Control (*n* = 4)**	**Ang II infusion (*n* = 8)**
HR (bpm)	375.32 ± 17.73	383.22 ± 15.53
LVESV (μl)	169.99 ± 17.25	123.87 ± 5.57^*^
LVEDV (μl)	520.72 ± 13.33	360.55 ± 38.05^*^
SV (μl)	350.74 ± 18.34	236.68 ± 33.58^*^
CO (ml/min)	131.34 ± 7.36	90.47 ± 13.47
EF (%)	67.38 ± 3.24	63.68 ± 3.07
E/A (mm/s)	1.26 ± 0.17	1.14 ± 0.05
E/E' (mm/s)	−17.72 ± 1.44	−28.08 ± 2.18^*^
IVSd (mm)	1.42 ± 0.05	2.09 ± 0.15^*^
LVPWd (mm)	1.70 ± 0.16	2.44 ± 0.12^*^
LV Mass (mg)	731.39 ± 23.04	889.15 ± 42.77^*^

* *p < 0.05. HR, heart rates; LVESV, left ventricular end-systolic volume; LVEDV, left ventricular end-diastolic volume; SV, stroke volume; CO, cardiac output; EF, ejection fraction; E/A, ratio of the early to late mitral inflow velocities; E/E′, ratio between early mitral inflow velocity and mitral annular early diastolic velocity; IVSd, Interventricular septal at the end of diastole; LVPWd, Left ventricular posterior wall at the end of diastole; LV, left ventricle*.

Primary isolates of fibroblasts from the heart are widely used as an *in vitro* model to study their signaling properties and cellular functions under defined experimental conditions. We have successfully established isolation and culture of adult rat ventricular fibroblasts in our laboratory and demonstrated the high purity of our fibroblast preparations (26). To demonstrate our findings that miRNA-1 is down-regulated in activated cardiac fibroblasts from Ang II-induced hearts also occur in cardiac fibroblasts in culture, we stimulated cardiac fibroblasts from normal untreated rats by Ang II or TGFβ, which are the two well-known pro-fibrotic agonists for fibroblast activation. As shown in Figure 1G, miRNA-1 was also down-regulated in response to Ang II or TGFβ stimulation in culture for 48 h and this down-regulation was more pronounced in TGFβ-stimulated fibroblasts. Together, these findings demonstrate that miRNA-1 is not only expressed in cardiac fibroblasts but is also markedly down-regulated upon cardiac fibroblast activation both *in vitro* and *in vivo*.

### MiRNA-1 Negatively Regulates Cardiac Fibroblast Proliferation

**Figure 2:**
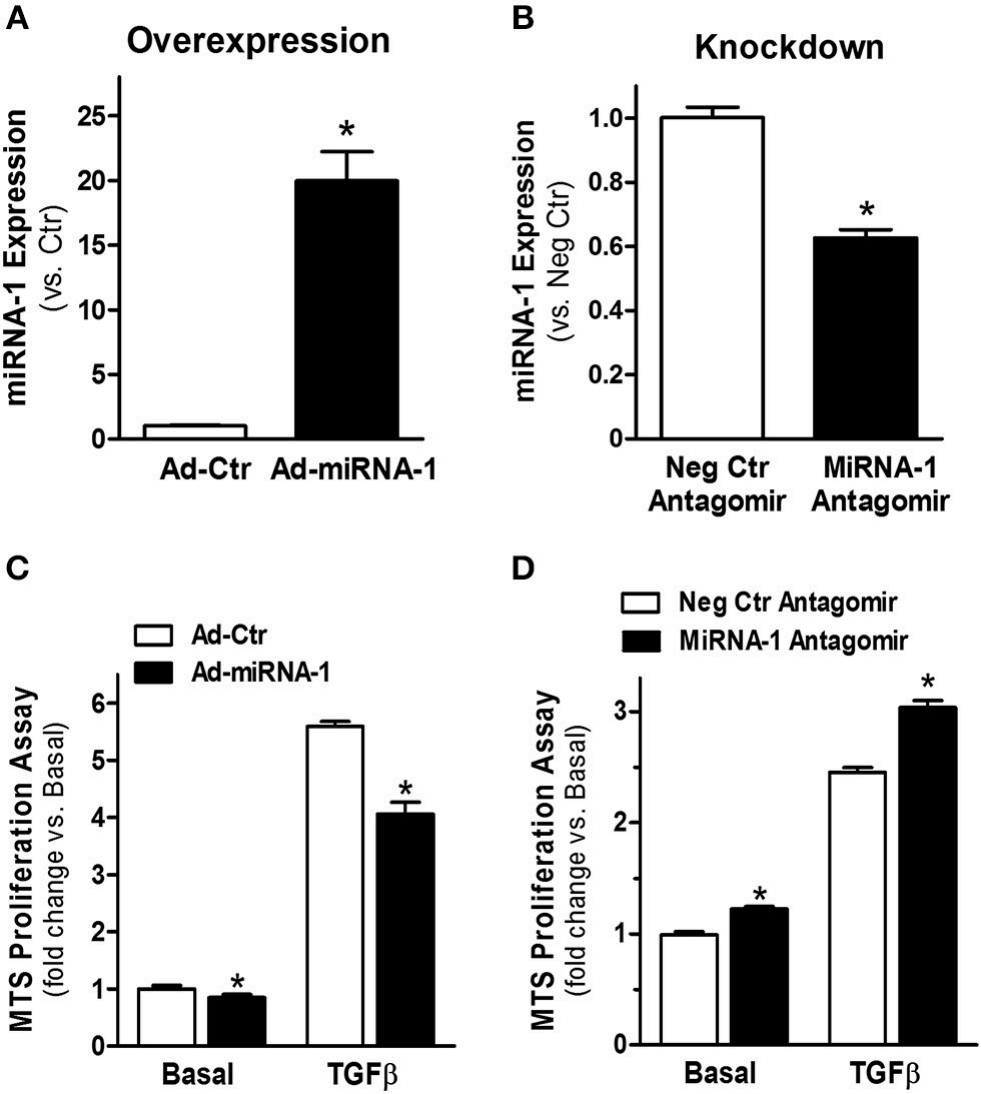
MiRNA-1 negative regulates cardiac fibroblast proliferation. **(A)** MiRNA-1 level in adult rat ventricular fibroblasts infected with Ad-Ctr or Ad-miRNA-1 (MOI 20) for 48 h. Data are normalized to U6 and expressed relative to controls. *n* = 6 each, ^*^ *P* < 0.05 vs. Ad-Ctr. **(B)** MiRNA-1 level in adult rat ventricular fibroblasts transfected with negative control (Neg Ctr) or miRNA-1 antagomirs for 48 h. Data are normalized to U6 and expressed relative to controls. *n* = 6 each. ^*^P < 0.05 vs. Neg Ctr antagomir. **(C)** Basal and TGFβ1 (10 ng/ml, 48 h)-induced cardiac fibroblast proliferation 72 h after Ad-Ctr or Ad-miRNA-1 infections (MOI 20). Data are expressed as relative changes to controls under basal conditions. *n* = 7 each. ^*^P < 0.05 vs. Ad-Ctr. **(D)** Basal and TGFβ1 (10 ng/ml, 48 h)-induced cardiac fibroblast proliferation 72 h after transfection with negative control (Neg Ctr) or miRNA-1 antagomirs. Data are expressed as relative changes to controls under basal conditions. *n* = 6 each. ^*^P < 0.05 vs. Neg Ctr antagomir.

To investigate the functional effects of miRNA-1 in cardiac fibroblasts, we overexpressed and knocked down miRNA-1 levels in cultured fibroblasts using adenovirus encoding miRNA-1 or chemically modified sequence-specific miRNA-1 antagomir, respectively. We did not observe any apparent changes in cell viability or morphology after adenoviral infection or antagomir transfection. TaqMan miRNA-1 assays were performed to assess the miRNA-1 levels. Compared to fibroblasts infected with Ad-Ctr, a 20-fold increase in miRNA-1 expression was achieved in cells infected with Ad-miRNA-1 at MOI 20 for 48 h (Figure 2A). Conversely, compared to cells transfected with negative control miRNA antagomir, we were able to achieve 41% reduction in endogenous miRNA-1 with the miRNA-1 antagomir (Figure 2B). To investigate the inhibitory effects of miRNA-1 on fibroblast proliferation, we then performed MTS cell proliferation assays. As shown in Figure 2C, miRNA-1 overexpression led to a 15% and 28% reduction of basal and TGFβ-induced fibroblast proliferation, respectively. Conversely, miRNA-1 downregulation promoted a 21% increase of fibroblast proliferation under basal conditions and a 25% increase in TGFβ-induced fibroblast proliferation (Figure 2D). Together, these gain- and loss-of-function studies demonstrate that miRNA-1 negatively regulates cardiac fibroblast proliferation.

### MiRNA-1 Directly Targets Cyclin D2 and CDK6

**Figure 3:**
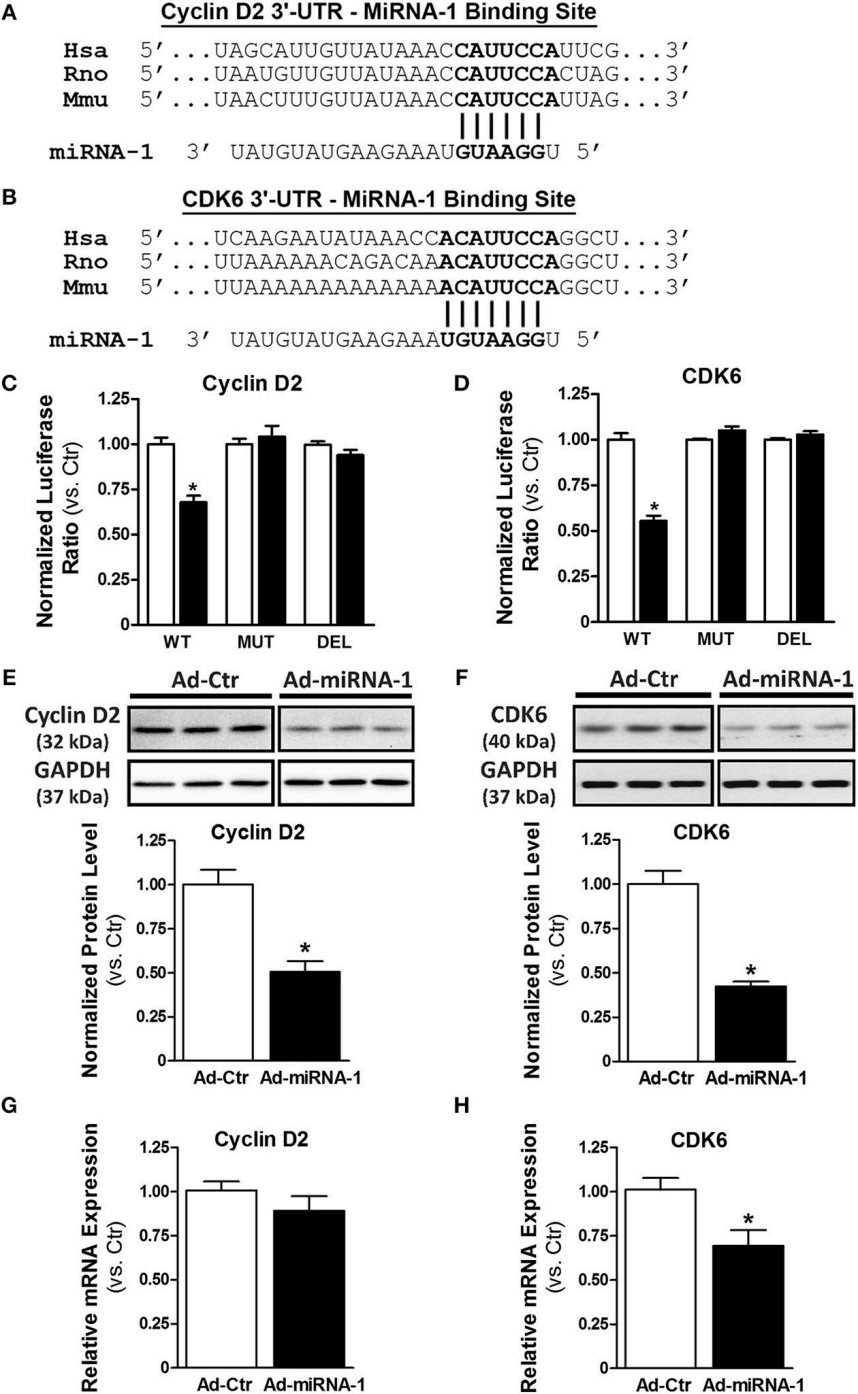
MiRNA-1 directly targets cyclin D2 and CDK6. **(A,B)** The sequences of putative miRNA-1 target sites as predicted by TargetScan and the conserved seed sequences are shown. **(C,D)** Normalized luciferase ratios in fibroblasts that were infected with Ad-Ctr or Ad-miR-1 (MOI 20) overnight, followed by transfection with plasmid constructs containing wild type (WT), mutation (MUT), or deletion (DEL) of the 3′-UTR binding site of the indicated putative target for 48–72 h. *n* = 6 each. ^*^ *P* < 0.05 vs. Ad-Ctr. **(E,F)** Western blots showing cyclin D2 and CDK6 expression in fibroblasts infected with Ad-Ctr or Ad-miRNA-1 (MOI 20) for 29 h. Representative images in each row are from the same membrane; spaces indicate removal of lanes with other conditions. Graphs showing quantitative densitometry and relative to Ad-Ctr. *n* = 6 each. ^*^ *P* < 0.05 vs. Ad-Ctr. **(G,H)** Real-time PCR analysis of cyclin D2 and CDK6 mRNA levels in fibroblasts infected with Ad-Ctr or Ad-miRNA-1 (MOI 20) for 48 h. Data are normalized to 18S and expressed relative to controls. *n* = 6 each. ^*^ *P* < 0.05 vs. Ad-Ctr.

To determine the molecular mechanism by which miRNA-1 negatively regulates cardiac fibroblast proliferation, we performed a bioinformatics search using TargetScan (available online at http://www.targetscan.org). Cyclin D2 and cyclin-dependent kinase 6 (CDK6), two important cell cycle regulators that initialize and promote cell cycle progression and cell proliferation, were identified as putative targets of miRNA-1. We next addressed whether cyclin D2 and CDK6 are direct target genes of miRNA-1 in cardiac fibroblasts using 3′-UTR luciferase reporter assays that are widely used to determine whether a miRNA directly binds to the 3′-UTR of its putative target genes. The predicted seed sequences of both cyclin D2 and CDK6 are evolutionarily conserved across species including mouse, rat, and human (Figures 3A,B). Overexpression of miRNA-1 led to a 32% and 45% reduction of normalized luciferase activity of the reporter gene of cyclin D2 and CDK6, respectively, in cardiac fibroblasts (Figures 3C,D), whereas these reductions were abrogated when the seed sequences of the 3′-UTR binding site were either mutated or deleted. Our results thus validate that cyclin D2 and CDK6 are direct targets of miRNA-1 in cardiac fibroblasts. We next evaluated the effects of miRNA-1 on protein expression of cyclin D2 and CDK6 in adult rat ventricular fibroblasts. As shown in Figures 3E,F, miRNA-1 expression markedly decreased the expression level of endogenous cyclin D2 and CDK6. To determine whether miRNA-1 regulates cyclin D2 and CDK6 protein expression via inhibition of translation or increase of mRNA degradation, we assessed cyclin D2 and CDK6 mRNA expression levels. As shown in Figures 3G,H, mRNA expression of cyclin D2 is not changed, suggesting that miRNA-1 may post-transcriptionally regulates its protein expression via inhibition of translation. In contrast, mRNA expression of CDK6 is down-regulated, indicating miRNA-1 may regulate its protein expression via mRNA degradation.

### MiRNA-1 Regulates Protein Expression of Cyclin D2 and CDK6 and Their Downstream Signaling in Cardiac Fibroblasts

**Figure 4:**
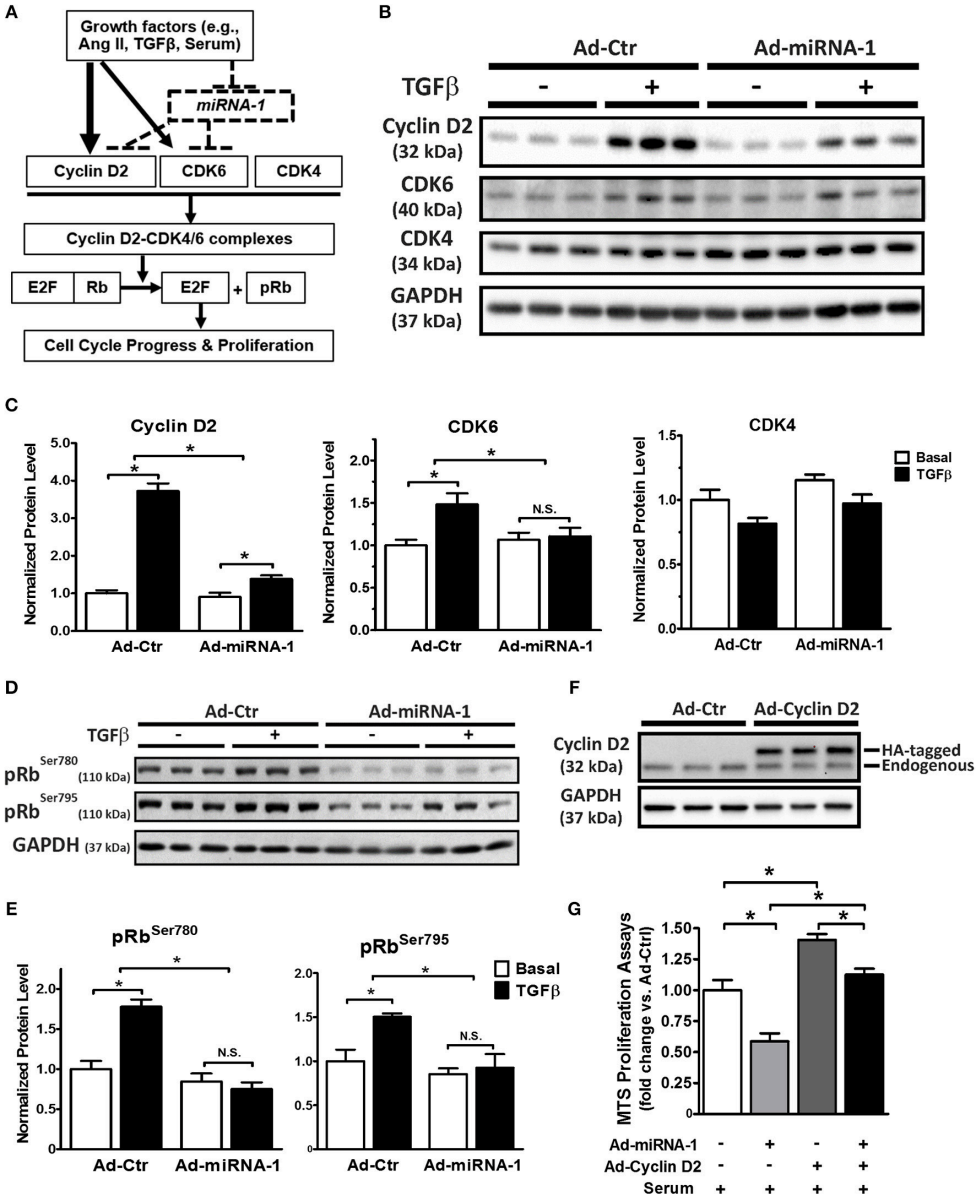
MiRNA-1 regulates cell cycle regulators and their downstream signaling in cardiac fibroblasts and overexpression of cyclin D2 blunts miRNA-1-mediated inhibition of cardiac fibroblast proliferation. **(A)** Scheme showing the working model of miRNA-1-mediated inhibition of cardiac fibroblast proliferation through targeting cyclin D2 and CDK6. **(B,C)** Western blots probed with the indicated antibodies. Cardiac fibroblasts were treated with or without TGFβ1 (10 ng/ml) for 48 h after Ad-Ctr or Ad-miRNA-1 infection (MOI 20) for 24 h. Graphs showing quantitative densitometry with relative changes to Ad-Ctr at basal condition. *n* = 6 each. ^*^ *P* < 0.05 for the indicated comparisons. **(D,E)** Western blots showing phosphorylation levels of Rb protein at Ser^780^ and Ser^795^ in cardiac fibroblasts that were treated with or without TGFβ1 (10 ng/ml) for 29 h after Ad-Ctr or Ad-miRNA-1 infection (MOI 20) for 24 h. Graphs showing quantitative densitometry with relative changes to Ad-Ctr at basal condition. *n* = 6 each. ^*^ *P* < 0.05 for the indicated comparisons. **(F)** Western blot showing overexpression level of HA-tagged cyclin D2 in cardiac fibroblasts after infection with Ad-Ctr or Ad-cyclin D2 (MOI 10) for 48 h. **(G)** Cardiac fibroblast proliferation in 2% FBS culture medium after infection with Ad-miRNA-1 (MOI 20) and/or Ad-Cyclin D2 (MOI 10) for 48 h. Ad-Ctr was used as control. Data are expressed as relative changes to controls with Ad-Ctr infection. *n* = 7 each. ^*^P < 0.05 for the indicated comparisons.

As illustrated in Figure 4A (solid lines), cell proliferation is often initialized by growth factors-mediated increase of protein expression of cyclin D (e.g., cyclin D2), which binds to the CDK4 and/or CDK6 to form cyclin D2/CDK4/6 complexes and subsequently promote cell cycle progression and proliferation by phosphorylation of the Rb protein and its dissociation from E2F transcription factors (31). Our data so far show that miRNA-1 is down-regulated in cardiac fibroblasts upon activation both *in vivo* and *in vitro* (Figure 1), that miRNA-1 negatively regulates cardiac fibroblast proliferation (Figure 2), and that miRNA-1 directly targets cyclin D2 and CDK6 in cardiac fibroblasts (Figure 3). We then delineated the regulatory mechanism of miRNA-1-mediated inhibition of cardiac fibroblast proliferation through targeting cyclin D2 and CDK6 (see the working model in Figure 4A) and their downstream signaling.

As shown in Figures 4B,C, miRNA-1 expression significantly inhibited TGFβ-induced protein expression of cyclin D2 and CDK6 in adult ventricular fibroblasts: TGFβ-induced rise in cyclin D2 expression was reduced from 3.7-fold in Ad-Ctr infected cells to 1.4-fold in Ad-miRNA-1 infected cells. Similarly, a 1.5-fold rise in protein expression of CDK6 in response to TGFβ stimulation was reduced to 1.1-fold over basal in Ad-miRNA-1 infected cells. CDK4, which is not a predicted target of miRNA-1, was not altered by miRNA-1 in its protein expression. We next assess the net inhibitory effect of miRNA-1 on cyclin D2/CDK4/6 complexes in cardiac fibroblasts using the phosporylation level of Rb protein at sites of Ser^780^ and Ser^795^ as a readout (32). As expected, TGFβ treatment increased Rb protein phosphorylation, and miRNA-1 expression markedly decreased TGFβ-induced Rb protein phosphorylation (Figures 4D,E). Together, these data suggest that miRNA-1 regulates cyclin D2 and CDK6 and their mediated signaling pathway for cell proliferation in cardiac fibroblasts.

To further determine whether the inhibitory effect of miRNA-1 on fibroblast proliferation is mediated by its regulation on cell cycle regulators, particularly cyclin D2 as the key cell cycle initiator, we also performed a rescue experiment by overexpression of miRNA-1 and/or cyclin D2 and assessed the effects on fibroblast proliferation. Figure 4F shows the efficiency of cyclin D2 overexpression, and miRNA-1 expression was same as reported in Figure 2A. As shown in Figure 4G, miRNA-1 overexpression led to a 42% reduction of 2% FBS-induced cardiac fibroblast proliferation and cyclin D2 overexpression promoted fibroblast proliferation by 40%. Importantly, when both miRNA-1 and cyclin D2 were overexpressed, the inhibition of miRNA-1 on cardiac fibroblast proliferation was blunted to 20%, suggesting cyclin D2 regulates miRNA-1-mediated fibroblast proliferation.

**Figure 5:**
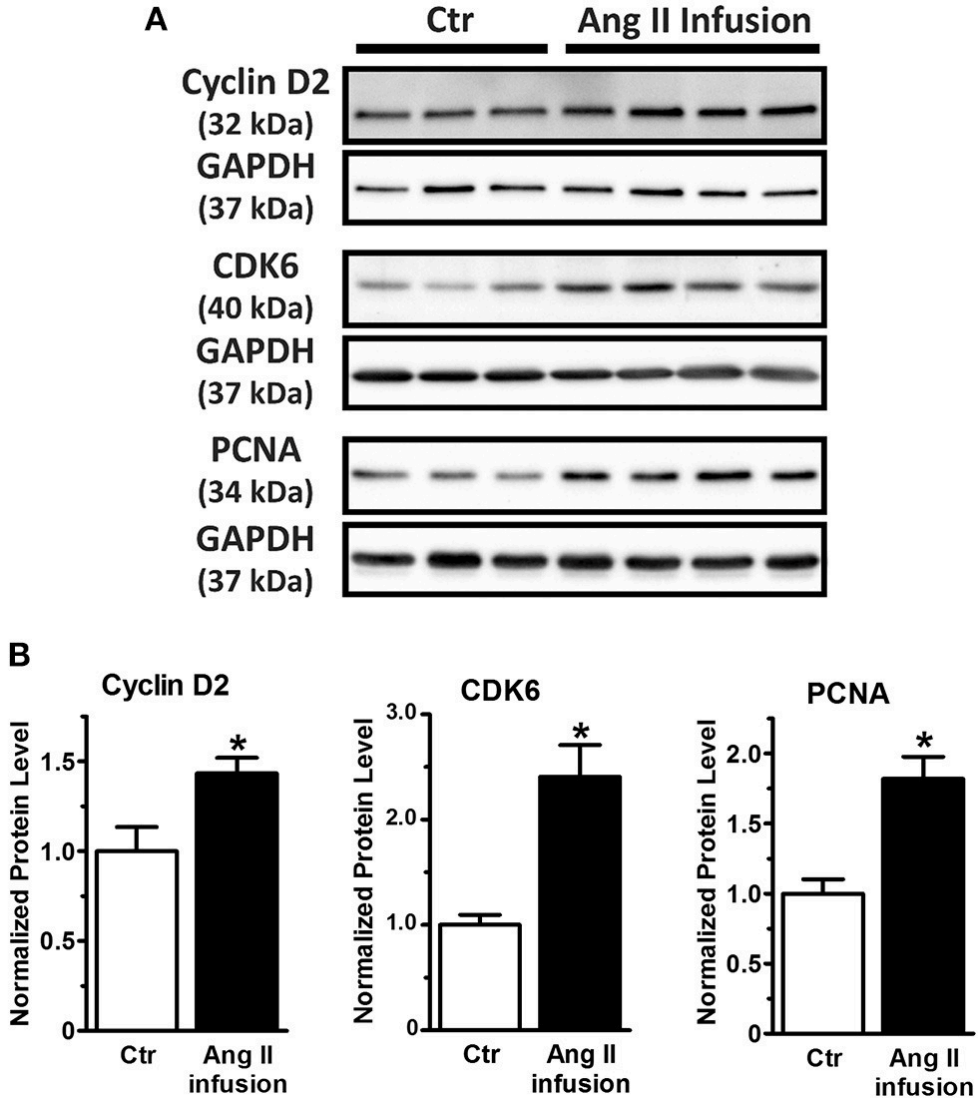
MiRNA-1's targets (cyclin D2 and CDK6) are up-regulated together with increase of cell proliferation marker PCNA in cardiac fibroblasts activated *in vivo* via subcutaneous Ang II-infusion for 4 weeks. **(A,B)** Western blots using cell lysates from freshly isolated cardiac fibroblasts from rats subjected to subcutaneous saline (Ctr, *n* = 3–5 rats) or Ang II infusion (*n* = 4 rats) for 4 weeks. Graphs showing quantitative densitometry of Western blots expressed as relative changes to Ctr. ^*^ *P* < 0.05 vs. Ctr.

Taken together, our study reveals miRNA-1 as a novel negative regulator of adult cardiac fibroblast proliferation *in vitro* that is at least partially mediated by direct targeting of cyclin D2 and CDK6 and their mediated cell cycle progression. Because miRNA-1 was markedly down-regulated in freshly isolated cardiac fibroblasts from Ang II-infused hearts (Figure 1F), we then examined the protein expression of cyclin D2 and CDK6 (as miRNA-1 targets) and PCNA (as a direct proliferation marker) in these fibroblasts. Compared to cardiac fibroblasts from control animals (Figure 5), freshly isolated cardiac fibroblasts activated by Ang II infusion for 4 weeks have a 1.5-fold and 2.4-fold increase of cyclin D2 expression and CDK6 expression, respectively. Importantly, a 1.8-fold increase of PCNA expression was also found in cardiac fibroblasts from Ang II infused group than that in control group, suggesting cardiac fibroblasts from interstitial fibrotic hearts induced by Ang II infusion have a significant increase in proliferation. These data thus not only validate our *in vitro* findings, but also point toward a potential important role of miRNA-1 in regulating cardiac fibroblast proliferation *in vivo*, which is beyond the scope of the present study and warrants future investigation.

## Discussion

MiRNA-1 has been long viewed as a muscle-specific miRNA and plays a critical role in myocardium and cardiomyocytes by controlling myocyte growth and rhythm (21). In this study, we discovered that miRNA-1 is expressed in cardiac fibroblasts and down-regulated upon fibroblast activation both *in vitro* and *in vivo*. We demonstrate that miRNA-1 negatively regulates cardiac fibroblast proliferation and that this effect is at least partially mediated by direct targeting of cell cycle regulators (i.e., cyclin D2 and CDK6) and the downstream signaling pathways. To our knowledge, this is the first report about the expression and regulation of miRNA-1 in cardiac fibroblasts that are either activated in culture or activated *in vivo* from hearts with interstitial/perivascular fibrosis. This is also the first report to show the role of miRNA-1 in regulating cardiac fibroblast function and signaling. Giving that cardiac fibroblasts are one of the major cell types in the myocardium and miRNA-1 has been reported as one of the major miRNA in regulating cardiomyocytes, this study expands our understanding of the regulatory roles of miRNA-1 in myocardium from cardiomyocytes to a major non-muscle cells.

### MiRNA-1 as a Novel miRNA in Cardiac Fibroblasts

Fibroblasts, together with the other cell types in the heart (myocytes, endothelial cells, and vascular smooth cells), are important determinants of structural, mechanical and electrical characteristics of the myocardium (33). It is well-recognized that fibroblasts are essential regulators of cardiac remodeling and fibrosis development and are therefore therapeutic targets (2, 6–8). However, efforts to develop therapies that specifically target fibroblasts are still at an early stage. Compared to traditional drug targets, miRNAs offer some novel mechanistic possibilities (9). Despite rapidly increasing insights into their roles in cardiac myocytes, the understanding of miRNAs in physiological and pathophysiological processes, particularly in cardiac fibroblasts, is still limited (34–36).

MiRNA-1 is a muscle-enriched miRNA, which represents up to 40% of all miRNA transcripts in adult mouse heart (37) and has been extensively studied in cardiac myocytes. Its expression is detected in the mouse heart around embryonic day 8.5 and gradually increases with a significant rise in the postnatal period (38). Expression and function of miRNA-1 in human vascular cells were also reported, including human endothelial cells (22) and human vascular smooth muscle cells (39). Dysregulation of miRNA-1 has been reported in several cardiac diseases from both animal models and human patients. MiRNA-1 is one of the earliest miRNAs down-regulated in cardiac hypertrophy induced by transverse aortic constriction-induced pressure overload in mice (28). Downregulation of miRNA-1 was also found in cardiac hypertrophy from genetic mouse models with overexpression of calcineurin (40) or AKT (41). Consistently, miRNA-1 levels are also decreased in patients suffering from left ventricle hypertrophy (28) or aortic stenosis with normal ejection fraction (42). Compared to hypertrophy, regulation of miRNA-1 expression in the failing hearts is more complex. For example, in ischemic and non-ischemic dilated cardiomyopathies, miRNA-1 was reported to be either up-regulated (43, 44) or down-regulated (42, 45), which may be due to sampling at different disease stages (e.g., transition from hypertrophy to dilated cardiomyopathy and heart failure). We should point out that in these studies miRNA analyses was performed on cardiac tissue, which reflects the total expression of miRNA-1 across all cell types in the myocardium. Because miRNA-1 is enriched in myocytes, its expression change observed in myocardium is likely majorly from cardiac myocytes. The expression and regulation of miRNA-1 in other cell types (e.g., cardiac fibroblasts) in the heart is unknown.

In this study, we first assessed the expression regulation of miRNA-1 in cardiac fibroblasts and myocytes in a chronic Ang II infusion-induced interstitial myocardial fibrosis rat model that also shows cardiac hypertrophy development. Cardiac fibrosis is one of the key components of the cardiac remodeling response in hypertensive cardiomyopathy (46). A crucial pathological feature in hypertension and eventually heart failure is the sustained activation of endogenous neurohormonal systems, particularly renin-angiotensin-aldosterone system (RAAS) (47, 48). It is well-documented that Ang II is the main effector peptide of RAAS. Infusion of exogenous Ang II has been a commonly used approach to model human hypertensive cardiomyopathy associated with increases in Ang II (49). We specifically chose an Ang II delivery rate of 555 ng·kg^−1^·min^−1^, because 555 ng·kg^−1^·min^−1^ in male Sprague-Dawley rats was shown to induce a 6-fold increase of plasma Ang II level in comparison to controls (50), which is in the same range as seen in heart failure patients [7-fold increase; (51)]. In line with the previous studies (see above), we found that miRNA-1 was down-regulated in hypertrophic myocytes from Ang II-infused hearts. Importantly, we discovered that miRNA-1 was also down-regulated in cardiac fibroblasts freshly isolated from the Ang II-infused hearts with myocardial interstitial fibrosis and, indeed, the down-regulation of miRNA-1 after 4-week Ang II infusion was more pronounced in cardiac fibroblasts than in cardiac myocytes (68 vs. 25%). Consistent with our observation from *in vivo* myocardial fibrosis model, our data showed that miRNA-1 is also downregulated in cardiac fibroblasts under pathologic stimulations by Ang II or TGFβ in culture. Together, these findings point to miRNA-1 as a potential player in the regulation of cardiac fibroblast function and signaling.

The underlying mechanism of miRNA-1 downregulation in cardiac fibroblasts upon stimulation is unclear and warrants future investigations. Several potential mechanisms could be involved. For example, activation of Janus kinase 2 (JAK2)/STAT3 pathway has been shown to suppress miRNA-1 expression by inhibiting miRNA-1 promoter activity in nasopharyngeal carcinoma (52). In cardiac fibroblasts, STAT3 is one of the first groups of transcriptional factors activated by external stress such as stimulation by cytokines or growth factors (53). Among them, Ang II is a well-documented inducer of JAK2/STAT3 signaling in cardiac fibroblasts (54, 55). Thus, we speculate that downregulation of miRNA-1 in cardiac fibroblasts in response to pathological stimulations, particularly Ang II, might be regulated by activation of JAK2/STAT3 pathway as well. Previous study also suggested that HDAC mediates miRNA-1 expression, which was indicated by the evidence that HDAC4 silencing induced miRNA-1 expression in human adenocarcinoma (A549) and human prostate cancer (DU-145) cell lines (52).

### MiRNA-1 as a Novel Regulator of Cell Proliferation in Cardiac Fibroblasts

Increased fibroblast proliferation in the myocardium is one of the major phenotypic changes of activated cardiac fibroblasts upon stimulation. In the present study, our data show that expression of exogenous miRNA-1 led to inhibition of basal and TGFβ-induced fibroblast proliferation. Conversely, knockdown of endogenous miRNA-1 significantly promotes fibroblast proliferation, indicating that endogenous miRNA-1 in cardiac fibroblasts exerts an inhibitory effect on fibroblast proliferation. It is indeed not easy to knock down miRNA-1 in adult cardiac fibroblasts by transfection. Following the manufacturer instruction, siPORT NeoFX transfection agent was used to transfect miRNA antagomirs in primary cells. We were able to achieve knockdown of endogenous miRNA-1 in cardiac fibroblasts by 40% without affecting cell viability or morphology. In our results, the increase of fibroblast proliferation upon knockdown of miRNA-1 was overall modest and we believe that this is at least in part related to the efficiency of miRNA-1 knockdown that can be achieved through transfection in adult rat cardiac fibroblasts.

Mechanistically, we demonstrate that miRNA-1 can directly target cyclin D2 and CDK6, which are two major cell cycle regulators, and exerts a combined effect by fine-tuning two proteins that depend on each other and act synergistically to promote cell cycle progression. Although miRNA-1 may have other targets that potentially also contribute to the observed inhibitory effect on fibroblast proliferation, direct targeting of cell cycle regulators (i.e., cyclin D2 and CDK6) by miRNA-1 would/should be a more direct mechanism for its inhibitory role on fibroblast proliferation, which is supported by the rescue experiments showing that the inhibitory effect of miRNA-1 on fibroblast proliferation is blunted by overexpression of cyclin D2. We also assessed the direct downstream signaling of cyclin D2 and CDK6 (i.e., net activity of cyclin D/CDK4/6 complexes), which is reflected by the phosphorylation status of Rb protein at sites of Ser^780^ and Ser^795^ (32). The Rb protein represses cell cycle progression by directly binding to the E2F transcription factors (56). In response to mitogenic stimuli, phosphorylation of Rb protein by cyclin D/CDK4/6 complexes is increased, which frees E2F transcription factors for their interaction with the transcription machinery of cells (31). Consistent with this notion, our data showed that TGFβ markedly induced phosphorylation of Rb protein at sites of Ser^780^ and Ser^795^ in fibroblasts, which was significantly diminished by miRNA-1 overexpression. Together, these findings suggest that miRNA-1 is a novel negative regulator of cardiac fibroblast proliferation, which is at least partially mediated by direct targeting of two cell cycle regulators.

Consistent with our findings in cardiac fibroblasts, cell cycle regulation by miRNA-1 was also suggested in cardiac myocytes: miRNA-1-2-null mice showed a 20% increase in the number of cardiac myocytes, and myocytes from these mice also displayed an increase in mitotic nuclei at postnatal day 10 that continued to varying degrees, even in the adult (21). Among the genes that were observed to be dysregulated in these mice, several were suspected to participate in the process of cell cycle regulation. Based on our findings here, it is conceivable that the mechanisms of direct targeting of cyclin D2 and CDK6 by miRNA-1 that we identified in cardiac fibroblasts might be applicable to cardiac myocytes and therefore could play a regulatory role in mediating myocyte proliferation capability and signaling. It would be interesting to ask whether the highest expression of miRNA-1 in cardiomyocytes also contributes to the mechanism of postnatal cardiomyocyte cell cycle withdrawal, which is still not fully understood.

## Conclusion and Outlook

The present study identifies miRNA-1 to be expressed in adult ventricular fibroblasts, where it acts as a novel negative regulator of adult cardiac fibroblast proliferation that is at least partially mediated by direct targeting of cell cycle regulators. This is the first step in our exploration of the functional role of miRNA-1 and the mechanism of its action in cardiac fibroblasts. MiRNA-1 likely has additional targets and may regulate other aspects of cardiac fibroblast biology, which warrants further investigation. For example, while the regulatory mechanism is under investigation, our unpublished data suggest that miRNA-1 also inhibits transformation of quiescent fibroblasts to activated myofibroblasts in culture. Recently, miRNA-1 was shown to directly target mitochondrial calcium uniporter in cardiomyocytes and subsequently regulate mitochondrial calcium contents and bioenergetics (57). Giving that mitochondria are also important organelles in cardiac fibroblasts, it is intriguing to know whether miRNA-1 can regulate mitochondrial signaling and function in cardiac fibroblasts as well. Our study also lays the ground for future investigations to delineate the physiological and pathophysiological role of miRNA-1 in regulating cardiac fibroblast function *in vivo*. A promising role of miRNA-1 in cardiac fibrosis was suggested by two *in vivo* studies with systemic alteration of miRNA-1 expression in the myocardium. As reported, mice with a global knockdown (~50%) of mature miRNA-1 showed cardiac dysfunction associated with significantly increased fibrosis (58). Consistently, global overexpression of exogenous miRNA-1 in the rat myocardium by adeno-associated virus with a CMV promoter showed a marked reduction of myocardial fibrosis in pressure overload-induced hypertrophy (59). However, because miRNA-1 levels were manipulated systemically (not cell type-specific) in both studies, it cannot be distinguished if the observed phenotypes in regulation of myocardial fibrosis development were due to regulatory effects of miRNA-1 in myocytes and/or fibroblasts. Thus, animal models with fibroblast-restricted miRNA-1 manipulation are needed to fully delineate the regulatory role of miRNA-1 in cardiac fibroblasts *in vivo*, which is beyond the scope of the present study.

## Data Availability

All datasets generated for this study are included in the manuscript and/or the supplementary files.

## Ethics Statement

This study was carried out in accordance with the recommendations of the Guiding Principles in the Care and Use of Vertebrate Animals in Research and Training. The protocol involving animals was approved by the Institutional Animal Care and Use Committee of Rhode Island Hospital. All efforts were made to minimize suffering.

## Author Contributions

PZ: conception and design. NV, MK, JM, HL, XL, and PZ: data acquisition and analysis. NV and PZ: data interpretation and drafting of the manuscript. PZ: revision of the manuscript.

### Conflict of Interest Statement

The authors declare that the research was conducted in the absence of any commercial or financial relationships that could be construed as a potential conflict of interest.
